# Friedelin Synthase from *Maytenus ilicifolia*: Leucine 482 Plays an Essential Role in the Production of the Most Rearranged Pentacyclic Triterpene

**DOI:** 10.1038/srep36858

**Published:** 2016-11-22

**Authors:** Tatiana M. Souza-Moreira, Thaís B. Alves, Karina A. Pinheiro, Lidiane G. Felippe, Gustavo M. A. De Lima, Tatiana F. Watanabe, Cristina C. Barbosa, Vânia A. F. F. M. Santos, Norberto P. Lopes, Sandro R. Valentini, Rafael V. C. Guido, Maysa Furlan, Cleslei F. Zanelli

**Affiliations:** 1 Instituto de Química, Univ. Estadual Paulista-UNESP, Rua Prof. Francisco Degni, 55, Quitandinha, Araraquara, SP 14800-060, Brazil; 2 Centro de Pesquisa e Inovação em Biodiversidade e Fármacos, Instituto de Física de São Carlos, Universidade de São Paulo, São Carlos, SP 13563-120, Brazil; 3 Faculdade de Ciências Farmacêuticas, Univ. Estadual Paulista-UNESP, Rod. Araraquara-Jaú km 1, Araraquara, SP 14801-902, Brazil; 4 Faculdade de Ciências Farmacêuticas, Universidade de São Paulo, Avenida do Café s/n, Monte Alegre, Ribeirão Preto, SP 14040-903, Brazil

## Abstract

Among the biologically active triterpenes, friedelin has the most-rearranged structure produced by the oxidosqualene cyclases and is the only one containing a cetonic group. In this study, we cloned and functionally characterized friedelin synthase and one cycloartenol synthase from *Maytenus ilicifolia* (Celastraceae). The complete coding sequences of these 2 genes were cloned from leaf mRNA, and their functions were characterized by heterologous expression in yeast. The cycloartenol synthase sequence is very similar to other known OSCs of this type (approximately 80% identity), although the *M. ilicifolia* friedelin synthase amino acid sequence is more related to β-amyrin synthases (65–74% identity), which is similar to the friedelin synthase cloned from *Kalanchoe daigremontiana*. Multiple sequence alignments demonstrated the presence of a leucine residue two positions upstream of the friedelin synthase Asp-Cys-Thr-Ala-Glu (DCTAE) active site motif, while the vast majority of OSCs identified so far have a valine or isoleucine residue at the same position. The substitution of the leucine residue with valine, threonine or isoleucine in *M. ilicifolia* friedelin synthase interfered with substrate recognition and lead to the production of different pentacyclic triterpenes. Hence, our data indicate a key role for the leucine residue in the structure and function of this oxidosqualene cyclase.

Terpenes are a special group of secondary metabolites. Their biosynthetic pathways begin with the condensation of isoprenyl units, isopentenyl diphosphate (IPP) and its isomer dimethylallyl diphosphate (DMAPP), which constitute the building blocks of terpenes with chemical skeletons ranging from 10 to 40 carbons. These compounds are present in prokaryotic and eukaryotic primary metabolism, although they are also present in eukaryotic secondary metabolism^1^.

Among terpenes, triterpenes are directly derived from the enzymatic cyclization of 30-carbon squalene in bacteria or from the cyclization of derived 2,3-oxidosqualene in eukaryotes and are catalysed by oxidosqualene cyclases (OSCs). OSCs are product specific and compete for the substrate oxidosqualene to produce triterpenes. The cyclization of oxidosqualene is initiated by the formation of a carbocation, which undergoes several rearrangements leading to the formation of a variety of compounds^2^ (Fig. 1). Among plant pentacyclic triterpenes, germanicol is the molecule with the smallest number of rearrangements, while friedelin 1 has the highest number^2^,^3^,^4^.

Triterpenes can play a role in membrane structural and hormonal functions, are the precursors of steroids (hopanoids in bacteria and cycloartenol in plants are the precursors to phytosterols, ergosterol in fungi, and cholesterol and steroid hormones in mammals), or can exhibit defensive properties in plants as pentacyclic triterpenes^5^,^6^,^7^.

The biological activities of friedelin have been extensively investigated. For example, the friedelin molecule has shown antimicrobial activity against the Gram-positive and -negative bacteria^8^ *Mycobacterium bovis, Mycobacterium tuberculosis*^9^, *Mycobacterium madagascariense*, and *Mycobacterium indicus pranii*^10^ as well as the ability to affect growth inhibition in *Candida* spp.^8^, *Trichophyton* and *Aspergillus niger*^11^. Additionally, friedelin has also shown vasodilation, anti-histaminic, anti-inflammatory, analgesic and antipyretic properties^12^; gastroprotective^13^, antioxidant and liver protective^14^ activities; and the ability to inhibit some cancer cell lines^15^.

Aside from its own biological activities, friedelin is claimed to be the precursor of antitumoural quinone methide triterpenoids, including maytenin and pristimerin in the Celastraceae and Hippocrateacea families^4^,^16^,^17^. It has been demonstrated that friedelin is synthesized after oxidosqualene cyclization in the leaves of *Maytenus aquifolium* and *Salacia campestris* and is transported to their roots where it is converted by oxidoreductases to maytenin and pristimerin^4^. Both molecules have been recognized for their anti-inflammatory^18^,^19^,^20^ and antimicrobial activities^4^,^21^,^22^,^23^,^24^ and are promising antitumour agents^25^,^26^,^27^.

Among the oxidosqualene cyclases (OSCs), the tetracyclic triterpene synthases lanosterol synthase and cycloartenol synthase are the most studied and have several structural features involved in the cyclization rearrangement specificity of their products^28^,^29^,^30^,^31^. Nevertheless, there is only one representative oxidosqualene cyclase (OSC) structure available in the protein data bank from *Homo sapiens*^32^ and one squalene cyclase from *Alicyclobacillus acidocaldarius*^33^. Compared to the tetracyclic triterpene synthases, less is known about the product specificity of the pentacyclic triterpene synthases, although some studies have been reported. For example, the N-terminal is indicated as the most important region for the specificity between lupeol synthase (from *Arabidopsis thaliana*) and β-amyrin synthase (from *Panax ginseng*)^34^. Tryptophan and leucine residues in the Met-Trp-Cys-Tyr-Cys-Arg (MWCYCR) motif from *Panax ginseng* β-amyrin synthase and the Met-Leu-Cys-Tyr-Cys-Arg (MLCYCR) motif from *Olea europaea* lupeol synthase have been identified as important for product differentiation. Moreover, the conserved tyrosine residue in the same motif has been determined as essential for the stabilization of one of the cation intermediates that produces the pentacyclic triterpenes. In contrast, a histidine is found in the analogous position in lanosterol and cycloartenol synthases and is suggested to be essential for the formation of tetracyclic triterpenes^35^.

To the best of our knowledge, the friedelin synthase gene has been cloned from only one species (*Kalanchoe daigremontiana*). Thus, the structural features related to its activity have not been addressed^3^. Therefore, to investigate the structural basis underlying the product specificity, we successfully cloned and expressed OSC genes from *Maytenus ilicifolia*. We identified a new friedelin synthase homologue and characterized its enzymatic activity. Moreover, mutagenesis studies conducted on leucine at position 482 (Leu482) and molecular modelling indicate the structural determinants that might be involved in product specificity and friedelin production.

## Results and Discussion

### Cloning of full-length cDNAs from *M. ilicifolia* encoding 2,3-oxidosqualene cyclases

Friedelin is one of the main triterpenes in *M. ilicifolia* leaves. To isolate the entire cDNA encoding friedelin synthase and the other 2,3-oxidosqualene cyclases expressed in these tissues, we employed a homology-based strategy to amplify the OSC core fragments, which were then extended using 5′- and 3′- Rapid amplification of cDNA ends (RACE). Degenerate primers were designed based on the conserved regions from plant OSCs found by multiple sequence alignments. OSC core fragments of approximately 750 bp were amplified from the total cDNA of *M. ilicifolia* leaves using degenerate primers. The fragments were sequenced and identified by a Basic Local Alignment Search Tool (BLAST) analysis. Two different OSC core sequences were identified, and these fragments were further extended in the 5′ and 3′ directions by RACE. Based on this strategy, three open reading frames (ORFs) for 2,3-oxidosqualene cyclases from *M. ilicifolia* leaves were identified. Sequence comparison using BLAST showed high similarity among the *M. ilicifolia* OSCs and related proteins, such as β-amyrin synthase, lupeol synthase and cycloartenol synthase, from various other plant species (Supplementary Table S1).

### Functional characterization of *M. ilicifolia* 2,3-oxidosqualene cyclases

To characterize the enzymatic activities of *M. ilicifolia* OSCs, their complete ORFs were cloned into the yeast expression vector pYES2, which is under the control of a galactose inducible promoter, and transformed into the *S. cerevisiae* strain VZL 1303 (generated as described in Materials and Methods section). The resulting *S. cerevisiae* VZL 1303 + pYES2-OSCs cells were induced to express the *M. ilicifolia* OSCs and used for the extract preparation of the triterpene products, which were analysed using gas chromatography and mass spectrometry (GC-MS). The chromatograms showed that each extract contained a single pentacyclic triterpene, which was not detected in the control with the empty vector (Fig. 2). The GC-MS analysis confirmed the production of plant triterpenes in *S. cerevisiae* by a comparison with a friedelin standard. Mass spectra of the detected compounds are presented in Supplementary Fig. S1. The two OSC ORFs were characterized as follows: (1) friedelin synthase from *M. ilicifolia (Mi*FRS–GenBank accession number KX147270) and (2) cycloartenol synthase from *M. ilicifolia, Mi*CAS1 (GenBank accession number KX147271). A Proton Nuclear Magnetic Resonance (^1^H-NMR) analysis confirmed that the compound obtained by the expression of the recombinant *Mi*FRS was friedelin. The carbon-13 Nuclear Magnetic Resonance (^13^C-NMR) spectroscopic data corroborated the presence of friedelin due to the following characteristic signals: (1) a carbonyl group at δ 213 (C-3) and (2) a methyl group with a gamma protection effect at δ 6.8 (C-23)^36^,^37^. These signals were confirmed using heteronuclear multiple bond correlation (HMBC) spectra, which showed cross peaks between C-3 (δ 213.3) and H-2 (δ 2.38). ^1^H shifts and ^13^C characteristic signals for friedelin are presented in Supplementary Table S2. Although the chromatographic profile of yeast expressing *Mi*FRS and *Mi*CAS1 are not identical to the one from empty vector (pYES2) expression, the other peaks identified do not correspond to OSC products. On the other hand, they were identified as other compounds of the ergosterol pathway, like 4,4-dimethylzymosterol (at 30.5 min).

### Friedelin synthase is the most expressed OSC sequence in *M. ilicifolia* leaves

To compare the expression of the cloned OSCs to the presence of triterpenes in leaves, we used real-time quantitative reverse transcription PCR (qPCR) to determine their relative mRNA levels during the year. As shown in Fig. 3, the relative *Mi*FRS mRNA levels were higher than the other OSC throughout the year, although the levels significantly decreased from summer to spring. Interestingly, despite displaying relatively lower expression levels, the expression of the *Mi*CAS1 gene increased as *Mi*FRS gene expression declined.

High relative expression levels for *Mi*FRS were expected throughout the year (Fig. 3a) because friedelin is the major triterpene present in *M. ilicifolia* leaves (Fig. 3b)^38^. Cycloartenol 7, a precursor of phytosterols, was not detected in the chromatograms because it is a phytosterol precursor, even though the cycloartenol synthase cloned sequence was expressed during the year. The phytosterol already described in *M. ilicifolia* is stigmasterol^39^,^40^, which cannot be seen in the chromatogram of the leaves because the methodologies we used did not allow the simultaneous identification of stigmasterol and friedelin.

### The triterpene synthases from *M. ilicifolia* have representative OSC features

Compared with other functionally characterized OSC sequences deposited in GenBank, the OSCs cloned in this study cluster into the following two main groups: (1) pentacyclic triterpene synthases and (2) cycloartenol synthases. Using the neighbour-joining method, the analysis showed that the *Mi*FRS enzyme is a unique sequence and does not cluster with the friedelin synthase from *Kalanchoe daigremontiana (Kd*FRS) that is currently available. This observation is common among OSCs with the same function but from different species^41^ (Fig. 4). As previously described, friedelin, glutinol and lupeol synthases from *K. daigremontiana (Kd*FRS, *Kd*GLS and *Kd*LUS) form one branch, while taraxerol synthase from the same species (*Kd*TAS) is closely related to β-amyrin synthases from *Betula platyphylla* (BPY) and from *Panax ginseng* (PNY1 and PNY2)^3^. Nevertheless, the *Mi*CAS1 enzyme clusters closely to the cycloartenol synthases clade. The predicted general protein features of the OSCs cloned in this study are shown in Supplementary Table 1. After multiple global alignments, *Mi*FRS showed high identity (65–74%) to several β-amyrin synthase sequences. *Mi*CAS1 is approximately 80% identical to other cycloartenol synthases.

Multiple sequence comparison analyses of the *M. ilicifolia* cloned ORFs identified the conserved superfamily domains squalene cyclase domain subgroup 1 (SQCY_1) and isoprene-C2-like reductase (ISOPREN_C2), which are present in class II terpene synthases, including OSCs. All of the sequences showed the presence of the Asp-Cys-Thr-Ala-Glu (DCTAE) motif, which contains the catalytic aspartic acid residue (initiator of oxidosqualene cyclization-substrate carbocation formation)^42^,^43^. They also showed the four QW motifs (conserved motifs rich in aromatic amino acids, starting with Q-Gln and ending with W-Trp), which are suggested to be important for enzyme structure maintenance^3^,^44^. The presence of the characteristic Gly-Tyr-Asn (GYN) residues and the conserved Met-Trp-Cys-His-Cys-Arg (MWCHCR) motif from cycloartenol synthases as well as the Ser-Phe (SF) residues and conserved Met-X-Cys-Y-Cys-Arg (MXCYCR) motif from pentacyclic triterpene synthases at the predicted positions are in agreement with the functional classification of *Mi*CAS1 and *Mi*FRS, respectively^34^,^35^ (Fig. 5).

### The leucine residue closely upstream to the DCTAE motif plays an important role in the activity of friedelin synthase

Global alignment of *Mi*FRS with *Kd*FRS showed approximately 65% identity, which is similar to the identity shared between *Mi*FRS and other triterpene synthases (Supplementary Table 1). Nonetheless, when *Mi*FRS is compared with other OSCs, it shows some features that are not present in the other investigated sequences. It is worth noting that a leucine residue is present two positions upstream of the DCTAE motif in *Kd*FRS (Leu483) and *Mi*FRS (Leu482), whereas the corresponding position is substituted with a valine residue in lanosterol, β-amyrin and lupeol synthases and an isoleucine residue in cycloartenol synthases (Supplementary Fig. S2). Interestingly, a leucine residue is present in *K. daigremontiana* glutinol synthase (*Kd*GLS), which also produces friedelin as a minor product^3^, and in shionone synthase, an OSC that produces a tetracyclic triterpene that also contains a cetonic group in the A-ring^45^.

To better understand the contribution of Leu482 to *Mi*FRS friedelin synthase activity, we substituted the leucine residue with valine, threonine or isoleucine and evaluated the biological activities of the OSC mutants expressed in yeast. The resulting triterpenes from the modified enzymes were extracted from yeast and evaluated by GC-MS. As shown in Fig. 6, the substitution of leucine with valine (Leu482Val mutant) did not only abolish the production of friedelin but also enabled the production of β-amyrin, which is not observed in wild-type *Mi*FRS. However, the replacement of the leucine with threonine (Leu482Thr) led to the production of β-amyrin only. Finally, the substitution of leucine with isoleucine (Leu482Ile) did not impact friedelin production. Therefore Leu482, which is located close to the catalytic aspartate in the DCTAE motif, is described here as being important for friedelin production.

### Structural analysis

To shed light on the protein structural features related to friedelin synthase activity, the mutations were mapped onto the three-dimensional structure of *Mi*FRS. We generated a homology model based on human lanosterol synthase (Protein Data Bank ID 1W6K) (Supplementary Fig. S3). The globular monomeric structure of *Mi*FRS contains 771 residues and a calculated root-mean-square-deviation (RMSD) of 0.3 Å with the aligned Cα coordinates for *H. sapiens* homologous oxidosqualene cyclase 1W6K. The mainly alpha barrel topology of *Mi*FRS encompasses 21 alpha-helices (comprehensive structural and functional annotations for genome sequences CATH 1.50.10.20). The model also contains a squalene cyclase subgroup 1 domain (SQCY-residues 68–709).

**Figure 1:**
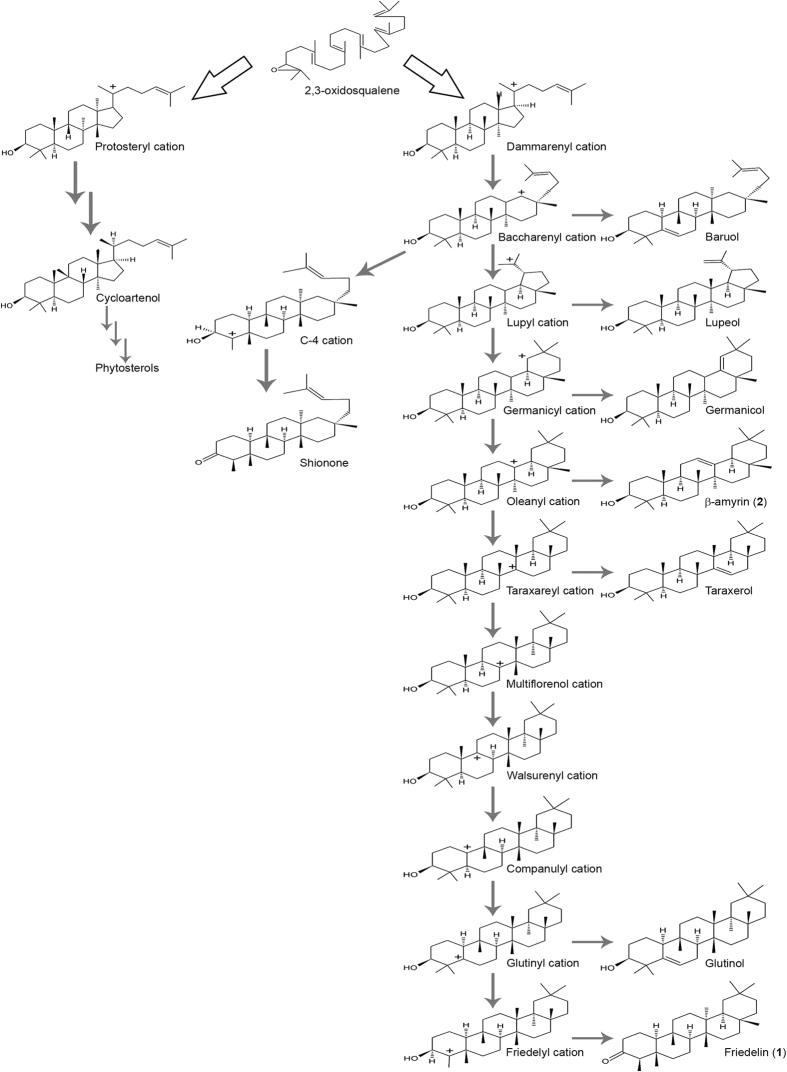
The proposed carbocation rearrangements for friedelin 1 formation. The oleanyl cation is a common branch for friedelin 1 and β-amyrin 2 formation.

**Figure 2:**
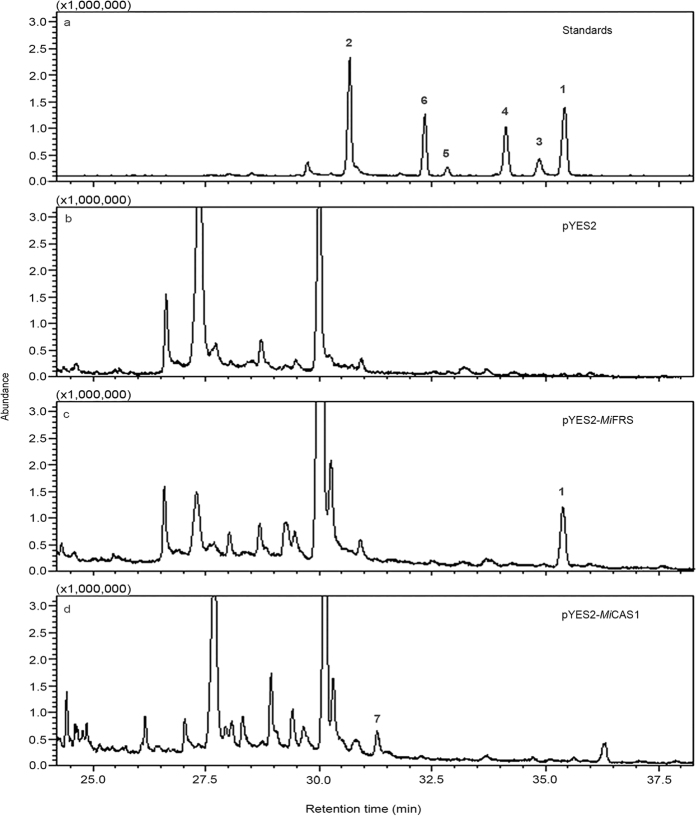
The characterization of *M. ilicifolia* friedelin and cycloartenol synthases using heterologous expression in *S. cerevisiae*. Total ion chromatograms (TIC) for (**a**) the standard compounds, including friedelin 1, β-friedelanol 3, α-amyrin acetate 4, β-amyrin acetate 5, epitaraxerol 6, and β-amyrin 2, used in this analysis and for the *S. cerevisiae* VZL 1303 extracts harbouring (**b**) empty vector pYES2; (**c**) vector carrying the coding sequence of friedelin synthase from *M. ilicifolia*, pYES2-*Mi*FRS; and (**d**) vector carrying the coding sequence of cycloartenol synthase 1 from *M. ilicifolia*, pYES2-*Mi*CAS1. The mass spectra with the fragmentation patterns for friedelin 1 and cycloartenol 7 are shown in Supplementary Fig. 1.

**Figure 3:**
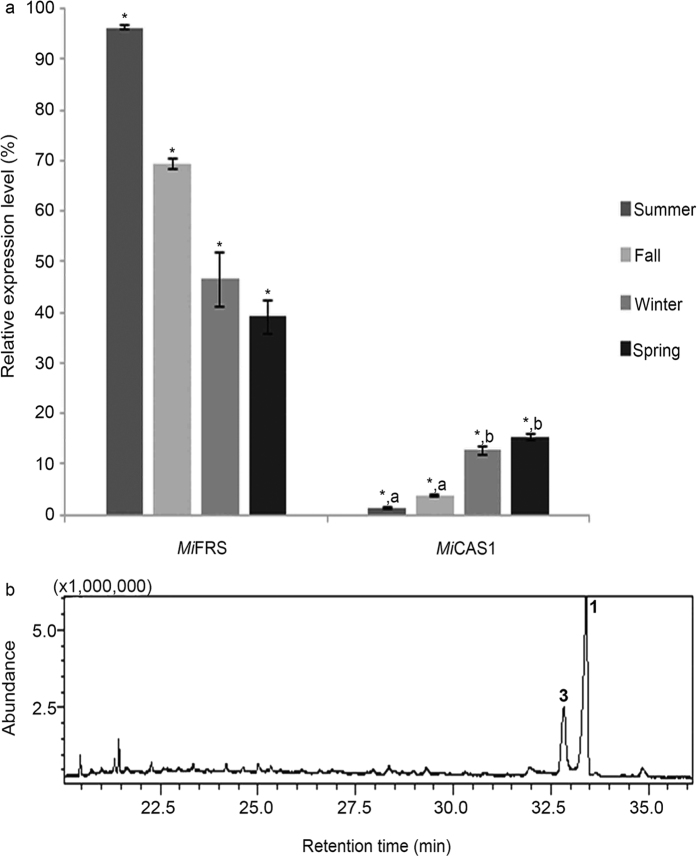
The relative expression levels of the OSCs cloned during different seasons in the year and triterpenes present in the leaves of *M. ilicifolia*. (**a**) The expression level of each gene was determined from RNA extracted from the leaves by qPCR in triplicate. A statistically significant difference is indicated by one asterisk (*) on the top of the bar. Bars with the same letter denote an absence of a statistically significant difference between each other on the expression of the genes in the respective season after analysis by Tukey’s test (P < 0.05). That is important to observe that the expression level of the gene encoding friedelin synthase is statistically different during the year and it is also statistically higher than the expression level of the gene encoding *Mi*CAS1. (**b**) TIC for the leaves of *M. ilicifolia*. Note for the prevalence of the triterpene friedelin 1 and the secondary β-friedelanol 3.

**Figure 4:**
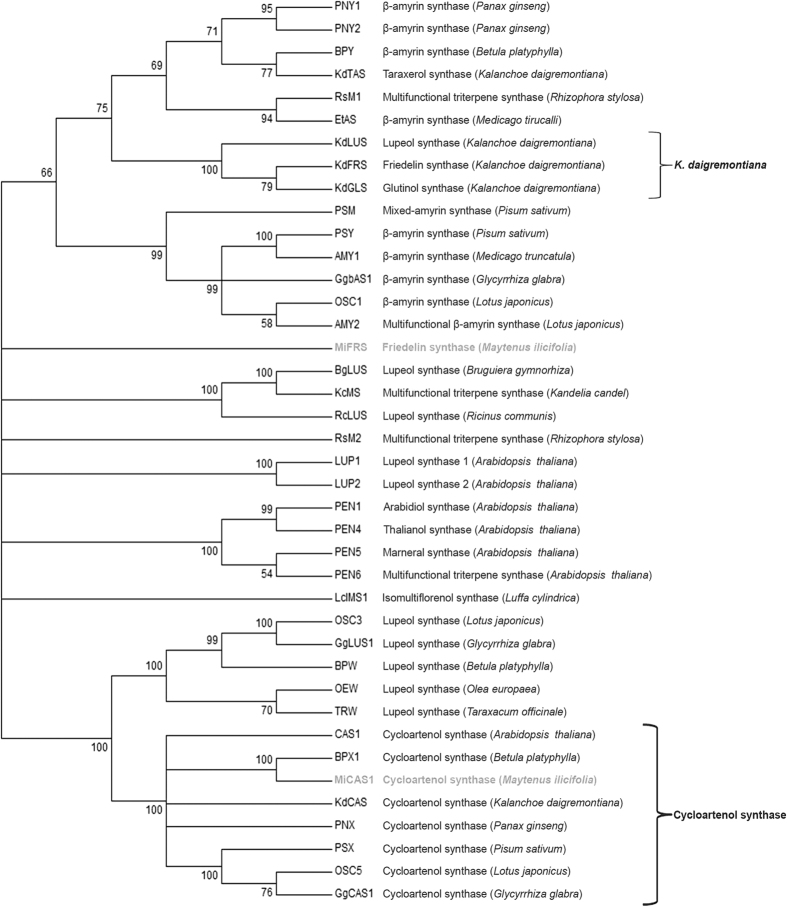
The phylogenetic analysis comparing the three cloned OSCs from *M. ilicifolia* leaves (in grey) with previously deposited OSCs from other plants. Although the *Mi*FRS and *Kd*FRS enzymes have the same functional characterization, the enzymes did not closely cluster. The GenBank data for the sequences used for this analysis are given in Supplementary Table 4.

**Figure 5:**
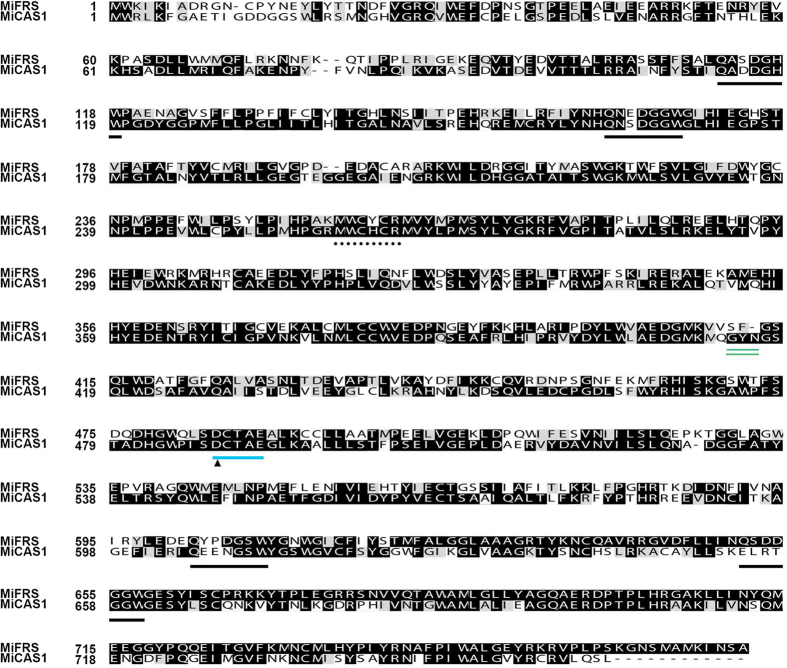
The amino acid sequences of the three OSCs cloned from *M. ilicifolia* leaves. Four conserved QW motifs and the catalytic site DCTAE are underlined, indicating the acids involved in substrate carbocation formation. Other conserved residues in the OSCs are MXCH/YCR, which are delimited by a dashed line, and the residues GYN and SF that differentiate tetracyclic to pentacyclic synthases, which are double underlined.

**Figure 6:**
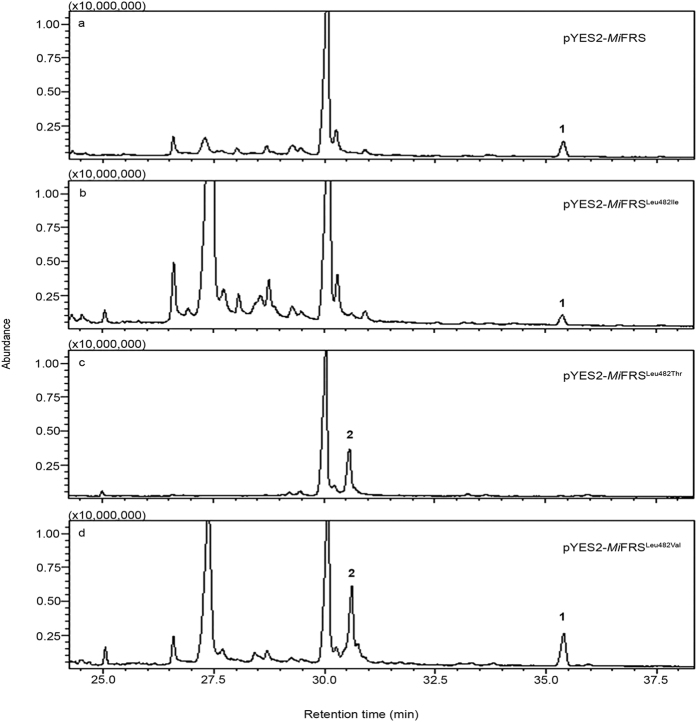
The TICs for the *Mi*FRS mutant Leu482 chloroform extracts expressed in yeast. (**a**) TIC of yeast extract harbouring pYES2-*Mi*FRS wild-type, (**b**) pYES2-*Mi*FRS^Leu482Ile^ mutant, (**c**) pYES2-*Mi*FRS^Leu482Thr^ mutant and (**d**) pYES2-*Mi*FRS^Leu482Val^ mutant. Note the absence of the peak corresponding to friedelin in the pYES2-*Mi*FRS^Leu482Thr^ mutant. The mass spectra fragmentation of the pentacyclic triterpene compounds is shown in Supplementary Fig. 1.

**Figure 7:**
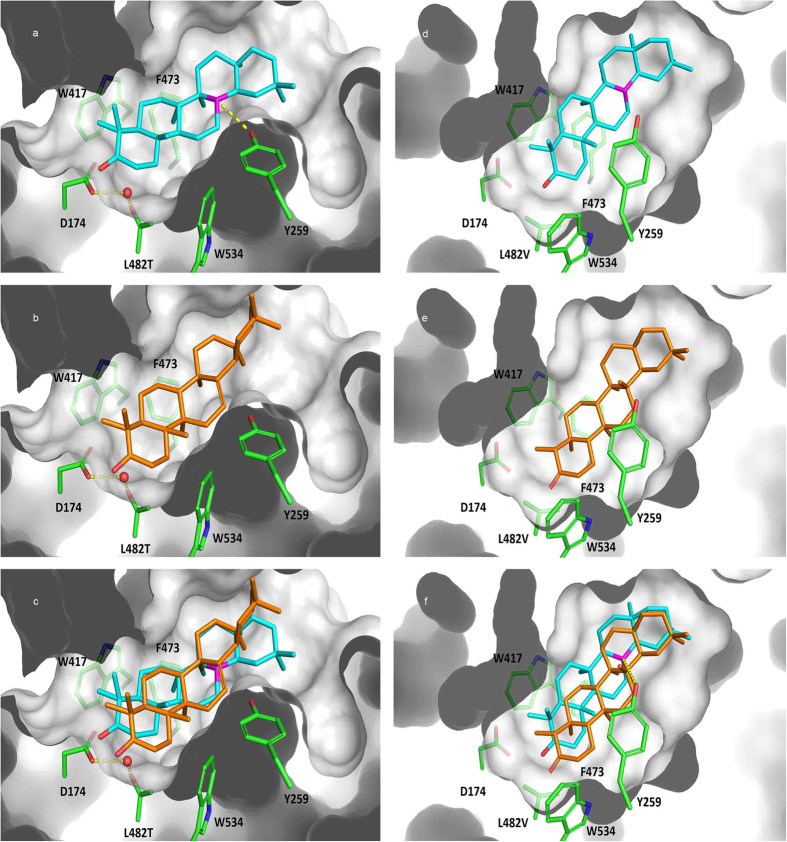
Modelled binding mode and poses overlays within *Mi*FRS mutated. (**a**) Modelled binding mode of oleanyl cation (cyan), (**b**) β-amyrin (orange) and (**c**) poses overlay within *Mi*FRS^Leu482Thr^ mutant binding site: the water molecule is indicated as red sphere, and polar interaction between Tyr259 and oleanyl cation (magenta) is indicated as yellow dashed lines. (**d**) Modelled binding mode of oleanyl cation (cyan), (**e**) friedelin (orange) and (**f**) poses overlay within *Mi*FRS^Leu482Val^ mutant binding site: polar interaction between Tyr259 and oleanyl cation (magenta) is indicated as yellow dashed lines.

To evaluate the impact of the changing Leu482 from a structural perspective, we considered previously proposed carbocation rearrangements in this analysis^2^ in which the oleanyl cation, a less rearranged species generated after oxidosqualene cyclization, is the closest common precursor of both β-amyrin and friedelin (as shown in Fig. 1). In this context, the Leu482Thr mutant would cause recruitment of a water molecule to the active site (Fig. 7a) due to the polar and smaller steric hindrance features related to the leucine residue in wild-type *Mi*FRS. In this site, the water molecule could stabilize the catalytic Asp174 residue and displace the oleanyl cation by positioning its C-ring closer to Tyr259 (Fig. 7a). The structural rearrangements within the active site may lead to carbocation stabilization, which ultimately leads to the formation of the unsaturated bond present in the β-amyrin ring (Fig. 7b). Additionally, the displacement induced by Leu482Thr places the A-ring closer to the hydrophobic side chains of Trp417, Phe473 and Trp534, thereby blocking the rearrangement of the methyl groups in the structure observed in the friedelin A-ring (Fig. 7c).

In contrast, the Leu482Val mutant would allow more mobility for the oleanyl cation in the active site, which is suggested by the small energy variations calculated for the different positions predicted by docking. The higher mobility allowed by Leu482Val in the active site confers less stabilization for the oleanyl cation at the C-ring by Tyr259 (Fig. 7d) compared with Leu482Thr, resulting in a system that can proceed to convert the oleanyl cation into both β-amyrin and friedelin (Fig. 7e,f) according to the proposed rearrangements (as represented in Fig. 1).

It is important to note that the Leu482Ile mutant did not impact friedelin production. This lack of impact can be explained by the similar volume and hydrophobicity maintained between the Leu and Ile side-chains at this position. Interestingly, most cycloartenol synthases have an isoleucine residue in this position, while most triterpene synthases have a valine residue.

A mutational study with cycloartenol synthase from *Pisum sativum* (PSX) has shown that a single amino acid changing (Tyr118Leu) resulted in almost exclusively cucurbitadienol production, although the inverse mutation in the cucurbitadienol synthase from *Cucurbita pepo* (CPQ) did not led to cycloartenol formation but parkeol^46^. The speculative explanation of the observed production modification was based on size changes and cation-π stabilization in the active site. Likewise, our model indicated that β-amyrin is formed by friedelin synthase single mutant (Leu482) specifically to Val or Thr due to the stabilization of the oleanyl cation in the active site cavity. The relationship between stabilization in the active site and the number of rearrangements to form a triterpene was corroborated by another study that analysed the variety of tetra and pentacyclic triterpene formation by the β-amyrin synthase from *Panax ginseng* (EtAS) cyclizing different oxidosqualene analogs. No mutational evaluations were conducted in that study, but it was determined that the methyl-30 group is important to keep the proper interaction with the enzyme so it can form pentacyclic triterpenes^47^.

Recently, another study describing mutants of the β-amyrin synthase SAD1 from *Avena strigosa* identified that the mutation Cys563Tyr block initiation of cyclization^48^. This is the third residue involved with initiation of cyclization so far, since both catalytic aspartic acid and the highly conserved cysteine of the DCTAE motif have previously been demonstrated to be essential for enzymatic activity^49^,^50^. This cysteine residue 563 of SAD1 (564 in *Mi*FRS) is highly conserved and had been previously predicted by the study of the human lanosterol synthase crystal structure to contribute for catalytic aspartic acid function due to their close positioning^32^. Besides this finding, that work demonstrated that the Ser728Phe mutant in SAD1 β-amyrin synthase from *Avena strigosa*, and the corresponding substitution Thr729Phe in *At*LUP1 from *A. thaliana*, favoured the production of tetracyclic triterpenes instead of β-amyrin, a pentacyclic and more rearranged triterpene. Using protein modelling and docking analysis, as performed herein, that work predicted that, in the SAD1 β-amyrin synthase Ser728Phe mutant, the large aromatic side chain of phenylalanine in position 728 interferes with the Phe725, of the catalytic centre, resulting in an interruption of ring expansion of the dammarenyl cation and leading to the formation of truncated tetracyclic oxidosqualene cyclization products. Moreover, the SAD1-Ser728Phe and AtLUP1-Thr729Phe mutants preferentially accepts dioxidosqualene as a substrate when expressed in yeast and cyclizes this to epoxydammarane derivatives, which suggests that Ser728 also affects the ability of the possible OSC substrates to access the active site^48^.

The analysis of products and substrate preference of mutants of triterpene synthases and the prediction of the role of specific amino acid residues by homology modelling and docking studies is a powerful strategy to explore triterpene variety as well as to enable the production of new compounds by controlling cyclization rearrangements and the acceptance of different substrates by OSCs. Therefore, our mutational studies on friedelin synthase activity, summed to the previous ones, are interesting examples of how the different determinants and motifs that distinguish different OSCs are established. Further understanding of the structural determinants will also allow for the design of OSC enzymes with increased specificity and productivity.

## Materials and Methods

### Plant material

The *Maytenus ilicifolia* (Celastraceae) specimen is maintained at the School of Pharmaceutical Sciences, São Paulo State University, Araraquara, Brazil (accession number 00755 at “Herbário do Departamento de Plantas Medicinais da Universidade de Ribeirão Preto” (HPM-UNAERP)). Leaves from the *Maytenus ilicifolia* were harvested and immediately placed in liquid nitrogen for grinding. The leaves were collected during 2011 and 2012.

### OSC cloning

Total RNA was extracted from the leaves of *M. ilicifolia* using a RNeasy Plant Mini Kit (Qiagen, Hilden, Germany) with a Buffer RLC (based on guanidine hydrochloride). RNA quality was verified by electrophoresis on a 1.2% agarose gel with samples loaded in a formamide buffer^51^. The ratios of absorbance at 260/280 and 260/230 were also determined by spectrophotometry. The cDNA was synthesized with the High-Capacity cDNA Reverse Transcription kit (Applied Biosystems, Foster City, California, USA) and used for polymerase chain reaction (PCR). The cloning strategy followed methods already described in previous studies^3^,^52^ using degenerate oligonucleotide primers designed in OSC conserved regions (Supplementary Table 3) and gene-specific primers for the amplification of coding sequence inner fragments. The *Escherichia coli* strain DH10B was used for cloning purposes. The 3′- and 5′-System for Rapid Amplification of cDNA Ends (RACE, Invitrogen, Carlsbad, California, USA) were used according to the manufacturer’s protocols with gene specific primers for amplifying the ORF end fragments, which were approximately 450 bp and 550 bp, respectively. PCR was performed with *Taq* DNA Polymerase (Fermentas, Waltham, Massachusetts, USA), and the PCR products were separated by 0.8% agarose gel electrophoresis and extracted using the QIAquick gel extraction kit (Qiagen). The fragments from each step were prepared to be cloned into the pTZ57R/T vector (Fermentas) and were transformed into competent *E. coli* cells. Plasmids containing the DNA fragments were purified from transformed cells with the QIAprep spin miniprep kit (Qiagen), sequenced using a Genetic Analyser 3130 with the Big Dye Terminator v3.1 Cycle Sequencing Kit and purified with the Big Dye XTerminator Purification Kit (Applied Biosystems). The different sequences obtained were identified as OSCs with the nucleotide BLAST tool (http://blast.ncbi.nlm.nih.gov/Blast.cgi) and grouped according to their identity. Full-length ORFs were amplified using *M. ilicifolia* cDNA as the template and specific primers (supplementary Table 3) for the 5′ and 3′ ends. PCR was performed with Platinum *Pfx* DNA Polymerase (2.5 U/μL; Invitrogen) under the following conditions: (1) hot start at 94 °C for 2 min, (2) 30 cycles of 94 °C for 30 s, 55 °C for 45 s and 68 °C for 3 min, and (3) a final extension a 68 °C for 10 min. The resulting 2.4 kb PCR product was cloned and sequenced with M13 and other primers (Supplementary Table 2).

### OSC functional analysis

The *S. cerevisiae* strain VZL 1303 (*MAT***a**, *Δura3, Δhis3, Δleu2, ERG7-kan*^r^ (DAmP, Decreased Abundance by mRNA Perturbation)) used in this study was obtained in our laboratory by crossing the CEN.PK2 and *ERG7* DAmP (Thermo Scientific, Waltham, Massachusetts, USA) strains. The full length *Maytenus ilicifolia* OSC ORFs were cloned into the yeast expression vector pYES2 (Invitrogen), which is under the transcriptional control of galactose (*GAL1* promoter). The pYES2-*Mi*FRS and pYES2-*Mi*CAS1- plasmids were transformed into VZL 1303, and the resulting strains were grown in 0.5 L of synthetic complete medium without uracil (SC-U). Heterologous gene expression was induced by 2% galactose for 10 h and triterpene production was achieved in the presence of 0.1 M potassium phosphate containing 3% glucose for 24 h at 30 °C with shaking. Then, the cells were collected, and the lipids were removed by a saponification reaction with 25% KOH in 50% ethanol solution (50 mL) in reflux for 5 min. Triterpenes were extracted by liquid-liquid partition 3 times with 50 mL of hexane^3^,^53^ and dried.

### Chemical analysis of the heterologously produced isoprenoids

The extract (90 mg) was suspended in 1 mL of hexane, submitted to column chromatography over silica gel (500 mg), and eluted with hexane (4 mL) and chloroform (6 mL), yielding the following 3 fractions: (1) Hex1 (8 mg), (2) Hex2 (14 mg) and (3) CHCl_3_ (42 mg). This procedure was performed in triplicates to verify the robustness of the process and analysis. The chloroform fractions, which were shown to be enriched with triterpenes, were analysed by gas chromatography with mass detection (GC-MS) using a QP-2010 (Shimadzu, Kioto, Japan) with a DB-5MS column (30 m × 0.25 mm × 0.25 μm; Agilent Technologies, Santa Clara, California, USA). Gas chromatography was performed with the following specifications: (1) inlet temperature of 250 °C, (2) heating gradient from 200 °C to 290 °C (10 °C/min), (3) trap temperature of 250 °C, (4) interface temperature of 290 °C, (5) injection volume of 1 μL, (6) split ratio of 1:10, (7) flow gas at 1.03 mL/min, (8) ionization of EI 70eV and (9) detection interval of 50 to 500 m/z. The structures derived from the detected peaks were searched against the National Institute of Standards and and Technology (NIST) library and compared with the literature and available standards. Nuclear Magnetic Resonance (NMR) spectra were recorded on a de NMR Bruker Avance III 600 (14.1 T; Billerica, Massachusetts, USA) spectrometer with CDCl_3_ as solvent and tetramethylsilane (TMS) as reference.

### Chemical analysis of triterpenes in the leaves of *M. ilicifolia*

Fresh leaves of *M. ilicifolia* (5 g) were harvested, immediately placed in liquid nitrogen for grinding and extracted with hexane:ethyl acetate (8:2, v/v). The extract was dried, resuspended in 1 mL of chloroform, filtrated and analysed by gas chromatography using the same method as above.

### Tissue expression levels

Leaves were collected during four seasons in one year, and total RNA was extracted as described above. The samples were treated with DNase I (1 U/μL, Sigma-Aldrich, St. Louis, Missouri, USA) before cDNA synthesis with the High Capacity cDNA Reverse Transcription Kit. Quantitative real-time PCR (qPCR) was performed using the Power SYBR Green PCR master mix (Applied Biosystems). Gene-specific primers were designed to amplify fragments of approximately 160 bp, and the 40S ribosomal protein gene (GenBank accession number KX147273) was used as an endogenous control (Supplementary Table 3). After primer validation and optimization of the reaction, qPCR was conducted with the following conditions: (1) 50 °C for 2 min, (2) 95 °C for 10 min, and (3) 40 cycles of 95 °C for 10 s and 60 °C for 1 min. The analysis was conducted in triplicates for each of the four leaf OSC transcripts from each season in the year, and the results are expressed as the average relative expression. Statistical significance was calculated using an analysis of variance (ANOVA) and a Tukey’s test. P-values less than 0.05 were considered significant.

### Phylogenetic analysis

OSC sequences from different plants were recovered from NCBI using the protein Blast tool (Supplementary Table 4) and were aligned with the four sequences cloned in this study using ClustalW (http://www.ebi.ac.uk/Tools/msa/clustalw2/). A neighbour-joining tree was constructed with the Mega 5 software default parameters based on 1000 bootstrap replications.

### *In silico* protein analysis

The translated amino acid sequence for each cloned OSC was obtained by the ORF Finder software (http://www.bioinformatics.org/sms2/orf_find.html). Comparisons of the deduced amino acids were performed with the protein Blast tool. To ensure the identification of the OSC superfamily in the cloned sequences, conserved domains were analysed with the NCBI Conserved Domain Search tool (http://www.ncbi.nlm.nih.gov/Structure/bwrpsb/bwrpsb.cgi), and the pI and molecular weight (MW) were predicted at http://web.expasy.org/compute_pi/. The three-dimensional structures were modelled using the modeller interface ViTaMIn tool (http://github.com/gustalima/vitamin-stable) and the crystal structure of *Homo sapiens* lanosterol synthase (PDB ID 1W6K) as a template. The ligand-binding amino acid residues in *Mi*FRS were predicted using Autodock Vina^54^.

### Mutagenesis of friedelin synthases

The pYES2-*Mi*FRS plasmid was used as a template for site-directed mutagenesis by a single primer amplification^55^. The primers used to create the substitution mutant at leucine 482 (in *Mi*FRS) are presented in Supplementary Table 3. Single primer amplifications were conducted with Phusion High Fidelity DNA polymerase (2000 U/mL; New England Biolabs) using the following conditions: (1) hot start at 98 °C for 1 min, (2) 30 cycles of 98 °C for 10 s, 50 °C for 30 s and 72 °C for 5 min, and (3) final polymerization at 72 °C for 5 min. The single-stranded amplification products were mixed and annealed by gradually decreasing the temperature 10 °C/min from 98 °C to 37 °C. After *Dpn*I (20 U/μL; New England Biolabs) treatment, the plasmids were transformed into competent *E. coli* cells. Sequencing was employed to confirm the presence of the desired mutations. Finally, the plasmids were transformed into VZL 1303 cells, and the functions of the *Mi*FRS mutants were analysed as described above.

### Protein homology modelling

The 771 amino acid *Mi*FRS protein sequence was translated from its DNA sequence retrieved from GenBank (KX147270). NCBI protein BLAST, used to search for suitable templates, identified the human homologue oxidosqualene cyclase (PDB 1W6K). A *Mi*FRS model was built using the satisfaction of spatial restraints algorithm implemented in MODELLER 9v14^56^,^57^,^58^. The best model was selected by assessing the Ramachandran plots and MODELLER’s DOPE score.

### Oleanyl cation docking

An oleanyl cation was docked using the Lamarckian Genetic Algorithm (LGA) implemented in Autodock 4.2 and AutoDock Vina^54^,^59^. The aim of this study was to identify the structural changes promoted by mutations that would lead to changes in oleanyl cation proton rearrangement, which further led to the formation of beta-amyrin or friedelin. In this sense, several oxidosqualene cyclases available as PDB structures were investigated (PDB IDs 1W6J, 1W6K, 2SQC, 3SQC, and 1GSZ). The structural analysis revealed a conserved water in the active site, suggesting a role in molecular recognition. Therefore, the water molecule was used to evaluate the ligand-binding mode as well as the relative position between the oleanyl carbocation and Tyr259. The search grid was defined as a cube with a 12 Å edge centred at Trp612. The residues 4 Å from the centre of the box (Trp257, Tyr259, Val263, Val410, Trp417, Phe473, Leu482, Aps484, Cys485, Trp534, Met549, Leu552, Ile555, Phe728, Leu734, and Tyr736) were set as flexible. The 3D structure of oleanyl molecule was generated using standard geometric parameters available in MarvinSketch (Marvin 16.2.22), 2016, ChemAxon (http://www.chemaxon.com). The optimized conformation of the ligands was energetically minimized using MarvinSketch default parameters. The results were analysed using PyMOL 1.7.2.

## Additional Information

**Accession codes:** *Mi*FRS–GenBank accession number KX147270; *Mi*CAS1–GenBank accession number KX147271.

**How to cite this article**: Souza-Moreira, T. M. *et al*. Friedelin Synthase from *Maytenus ilicifolia*: Leucine 482 Plays an Essential Role in the Production of the Most Rearranged Pentacyclic Triterpene. *Sci. Rep.* **6**, 36858; doi: 10.1038/srep36858 (2016).

**Publisher's note**: Springer Nature remains neutral with regard to jurisdictional claims in published maps and institutional affiliations.

## Supplementary Material

Supplementary Information
