# Supplementary material for: Friedelin Synthase from Maytenus ilicifolia: Leucine 482 Plays an Essential Role in the Production of the Most Rearranged Pentacyclic Triterpene
Source: Sci Rep. 2016 Nov 22;6:36858. doi: 10.1038/srep36858 (PMC5118845; doi:10.1038/srep36858)
Supplement: Supplementary Information [file srep36858-s1.pdf]

Supplementary information

Friedelin Synthase from *Maytenus ilicifolia*:

Leucine 482 Plays an Essential Role in the Production of the Most Rearranged Pentacyclic Triterpene

Tatiana M. Souza-Moreira, Thaís B. Alves, Karina A. Pinheiro, Lidiane G. Felippe, Gustavo M. A. De Lima, Tatiana F. Watanabe, Cristina C. Barbosa, Vânia A. F. F. M. Santos, Norberto P. Lopes, Sandro R. Valentini, Rafael V. C. Guido, Maysa Furlan, Cleslei F. Zanelli

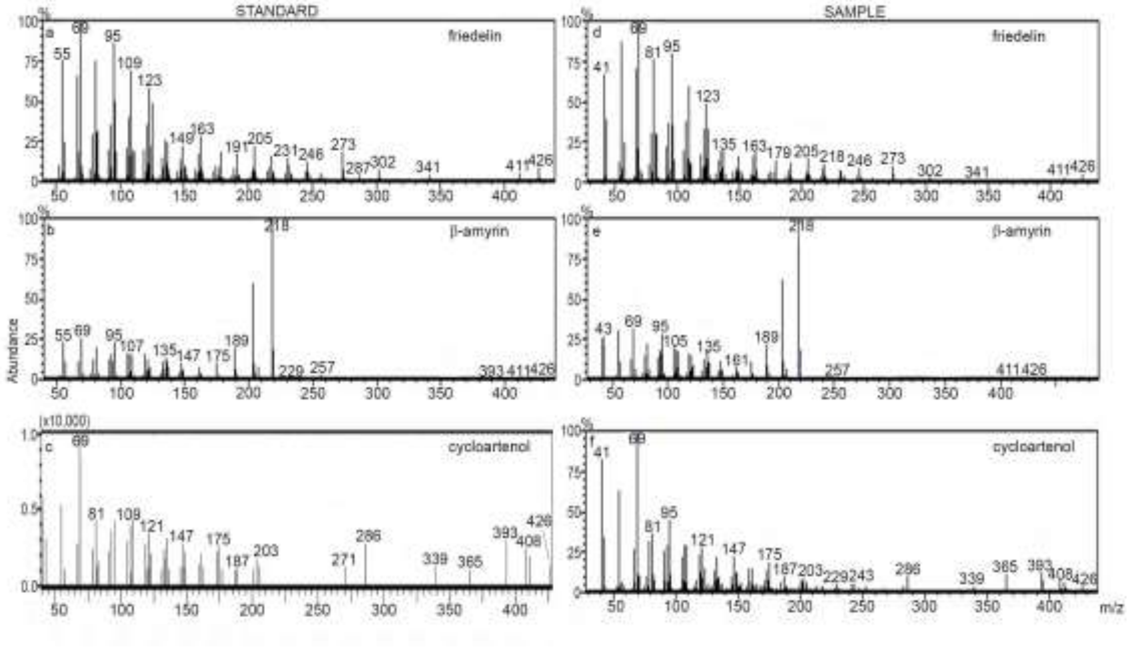

Supplementary Figure S1. The mass spectra for the triterpenoids analysed in this work. The fragmentation patterns of the standards (a) friedelin, (b) cycloartenol and (c) β-amyrin. Triterpene compounds produced by *Saccharomyces cerevisiae* heterologously expressing the plant oxidosqualene cyclases ((d) friedelin, (e) cycloartenol and (d) β-amyrin) show similar fragmentation patterns as standards. Mass spectrum of standard cycloartenol was obtained from the NIST08 library.

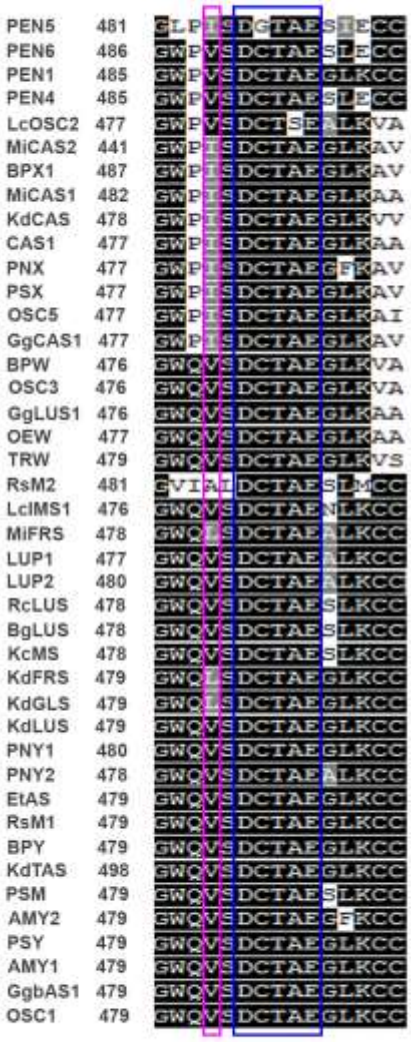

Supplementary Figure S2. The alignment of the different oxidosqualene cyclases (OSCs) around the Asp-Cys-Thr-Ala-Glu (DCTAE) conserved motif in the catalytic site. The leucine 2 positions before the DCTAE motif is present only in the friedelin-synthesizing enzymes. The alignment was performed using CLUSTALW (http://www.ebi.ac.uk/Tools/msa/clustalw2/). The oxidosqualene cyclase (OSC) identification and accession numbers are shown in Supplemental Table 3.

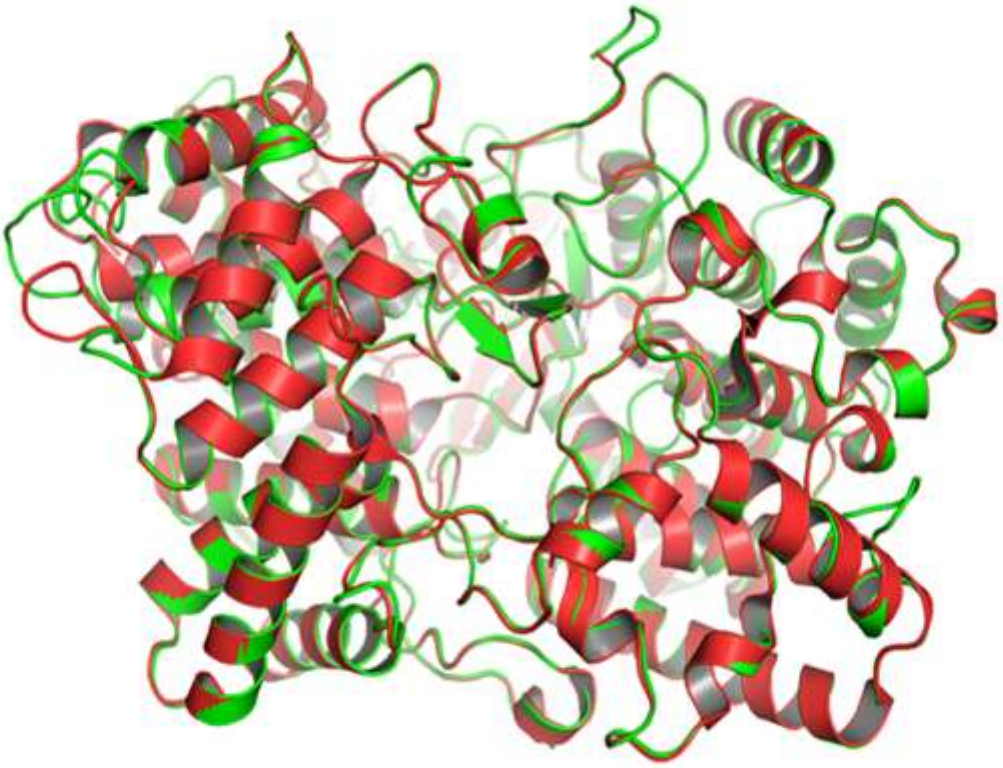

Supplementary Figure S3. The structural overlay of the friedelin synthase from *Maytenus ilicifolia* (*Mi*FRS, green, homology model) and human OSCs (red, template).

Supplementary Table S1. The features of the cloned OSC sequences and their translated protein sequences.

**ORF name**	***Mi*FRS**	***Mi*CAS1**
**ORF length**	2316 bp	2289 bp
**AA length**	771	762
**Predicted MW**	89.2 kDa	86.8 kDa
**Predicted pI**	6.04	5.88
**Identity to each other**	52% *Mi*CAS1	-
**Highest protein Blast identity**	65-71% β-amyrin synthases	72-81% cycloartenol synthases

*Mi*FRS = friedelin synthase from *Maytenus ilicifolia*; *Mi*CAS1 cycloartenol synthase 1 from *Maytenus ilicifolia*; bp = base pair; kDa = kilo daltons.

Supplementary Table S2. 1D NMR spectral data of friedelin ($CDCl_3$, 14.1T).

Position	^{13}C*	^{1}H
**1**	22.3	1.97 (m)
**2**	41.5	2.38 (m)
**3**	213.3	---
**4**	58.2	2.25 (m)
**5**	42.1	---
**6**	41.2	1.28 / 1.77 (m)
**7**	18.2	1.40 (m) / 1.50 (m)
**8**	53.1	1.40 (m)
**9**	37.4	---
**10**	59.4	1.53 (dd, *J* = 2.5, 12.5)
**11**	35.6	1.26 (m) / 1.48 (m)
**12**	30.5	1.19 (m) / 1.35 (m)
**13**	39.7	---
**14**	38.3	---
**15**	32.4	1.28 (m) / 1.50 (m)
**16**	36.0	1.36 (m) / 1.58 (m)
**17**	30.0	---
**18**	42.8	1.57 (d, *J* = 5.0)
**19**	35.3	1.21(m) / 1.38 (m)
**20**	28.1	---
**21**	32.7	1.53 (m) / 1.30 (m)
**22**	39.2	0.94 (m) / 1.51(m)
**23**	6.8	1.00 (d, *J* = 6.8)
**24**	14.6	0.73 (s)

Supplementary Table S2. Continuation

**25**	17.9	0.87 (s)
**26**	20.2	0.88 (s)
**27**	18.6	1.05 (s)
**28**	32.1	1.18 (s)
**29**	35.0	0.95 (s)
**30**	31.8	1.01 (s)

* Spectral data is in accordance to the literature[1].

Supplementary Table S3. The primers used in this study.

**Name**	**Sequence 5’- 3’**	**Target**	**Notes**
**Primers for cloning of the OSCs**			
Core amplification			
TS1F[2]	TTYGGNWGYCARNHRTGGGAT[1]	PTS	Degenerated
CS1F[2]	TAYAATGGMAGYCARYTVTGGGA[1]	CAS	Degenerated
CS1R[2]	TRRCTYTCHCYCCAHCCACC[1]	CAS/PTS	Degenerated
Core sequence extension			
BWN1F[2]	CARWAYRDDGAYGGNGGNTGGG[1]	all	Degenerated
GSP1R	GAGTAGGAGCTACTTCATCTGTGAG	MiFRS	
GSP3R	GACACAGACCATACTCTTCAAC	MiCAS1	
3’RACE			
GSP1F	GGAGGGCTAGCAGCAGCTGG	MiFRS	
GSP3F	AGGGGCTTGTAGCAGCTGGG	MiCAS1	
NGSP1F	TGTCAGGCTGTGCGCAGAG	MiFRS	Nested PCR
NGSP3F	AGCCTTCGTAAGGCTTGCGC	MiCAS1	Nested PCR
AUAP	GGCCACGCGTCGACTAGTAC	all	
5’-end RACE			
GSP5R	GCATCTTCGTCTGGTCCCACTC	MiFRS	cDNA
GSP7R	GTCCAAGATCCATTTCCTTCC	MiCAS1	cDNA
GSP9R	GCAAACATAAGTGAATGCTGTGGC	MiFRS	
GSP11R	CACCCTCTCCACCCTCAG	MiCAS1	
NGSP5R	GCAAACACTGTGCTGTGTCCC	MiFRS	Nested PCR
NGSP7R	AGCAACCTCAAAGTGACATAGTTC	MiCAS1	Nested PCR
AUAP	GGCCACGCGTCGACTAGTAC	all	
Full length cDNAs			
MiFRS1F	CGCGGATCCATCATGTGGAAGATAAAGATTGC	MiFRS	*Bam*HI site
MiFRS1R	CGGAATTCCGTGTAAGTGGACATATCATATGA	MiFRS	*Eco*RI site
MiCAS1F	CGCGGATCCGCGACAATGTGGAGGCTCAAATT	MiCAS1	*Bam*HI site
MiCAS1R	TGCTCTAGACAAATTCAAAGAGACTGTAATAC	MiCAS1	*Xba*I site
**q-RT-PCR**			
MI40SF	TCCTCTTGACCTGATTTGGG	40S	
MI40SR	ACTTTTGGGGGATGGACTTC	40S	
RTMiFRS1F	CCTCCAAGAGCCAAAAACTG	MiFRS	
RTMiFRS1R	CCATAGGGTTGAGCATCTCC	MiFRS	
RTMiCAS1F	GTTGGGGAACCTTTGGATG	MiCAS1	
RTMiCAS1R	GTTGCAAACCCACCATCAG	MiCAS1	
**Site-directed mutagenesis**			
L482IF	GATCATGGATGGCAA**ATT**TCTGATTGCACTG	MiFRS	
L482IR	CAGTGCAATCAGA**AAT**TTGCCATCCATGATC	MiFRS	
L482TF	GATCATGGATGGCAA**ACT**TCTGATTGCACTGC	MiFRS	
L482TR	GCAGTGCAATCAGA**AGT**TTGCCATCCATGATC	MiFRS	
L482VF	GATCATGGATGGCAA**GTT**TCTGATTGCACTG	MiFRS	
L482VR	CAGTGCAATCAGA**AAC**TTGCCATCCATGATC	MiFRS	

The letter F or R at the end of the name of each primer indicates if the primer is forward or reverse, respectively. PTS = pentacyclic triterpene synthase. CAS = cycloartenol synthase. GSP

= gene specific primer; NGSP = nested gene specific primer. Endonuclease restriction sites are underlined. Changed codons in the primers for site-directed mutagenesis are in bold.

Supplementary Table S4. The sequences used in the phylogenetic analysis with their GenBank accession number, species and function.

**Accession number**	**Species**	**Function**
AB257562.1	*Arabidopsis thaliana*	Arabidiol synthase (PEN1)
AY327541.1	*Arabidopsis thaliana*	Thalianol synthase (PEN4)
BT020456.1	*Arabidopsis thaliana*	Marneral synthase (PEN5)
AB274959.1	*Arabidopsis thaliana*	Multifunctional triterpene synthase (PEN6)
NM_179572.1	*Arabidopsis thaliana*	Lupeol synthase 1 (LUP1)
NM_106545.3	*Arabidopsis thaliana*	Lupeol synthase 2 (LUP2)
NM_126681.2	*Arabidopsis thaliana*	Cycloartenol synthase (CAS1)
AB263204.1	*Rhizophora stylosa*	Multifunctional triterpene synthase (RsM2)
AB289586.1	*Bruguiera gymnorhiza*	Lupeol synthase (BgLUS)
AB257507.1	*Kandelia candel*	Multifunctional triterpene synthase (KcMS)
DQ268869.1	*Ricinus communis*	Lupeol synthase (RcLUS)
AB058643.1	*Luffa cylindrica*	Isomultiflorenol synthase (LcIMS1)
AB037203.1	*Glycyrrhiza glabra*	β-amyrin synthase (GgbAS1)
AB181244.1	*Lotus japonicus*	β-amyrin synthase (OSC1)
AB034802.1	*Pisum sativum*	β-amyrin synthase (PSY)
AF478453.1	*Medicago truncatula*	β-amyrin synthase (AMY1)
AF478455.1	*Lotus japonicus*	Multifunctional β-amyrin synthase (AMY2)
AB034803.2	*Pisum sativum*	Mixed-amyrin synthase (PSM)

Supplementary Table S4. Continuation

AB009030.1	*Panax ginseng*	β-amyrin synthase (PNY1)
AB014057.1	*Panax ginseng*	β-amyrin synthase (PNY2)
AB055512.1	*Betula platyphylla*	β-amyrin synthase (BPY)
HM623868.1	*Kalanchoe daigremontiana*	Taraxerol synthase (KdTAS)
AB263203.1	*Rhizophora stylosa*	Multifunctional triterpene synthase (RsM1)
AB206469.1	*Medicago tirucalli*	β-amyrin synthase (EtAS)
HM623870.1	*Kalanchoe daigremontiana*	Friedelin synthase (KdFRS)
HM623869.1	*Kalanchoe daigremontiana*	Glutinol synthase (KdGLS)
HM623871.1	*Kalanchoe daigremontiana*	Lupeol synthase (KdLUS)
AB025343.1	*Olea europaea*	Lupeol synthase (OEW)
AB025345.1	*Taraxacum officinale*	Lupeol synthase (TRW)
AB181245.1	*Lotus japonicus*	Lupeol synthase (OSC3)
AB116228.1	*Glycyrrhiza glabra*	Lupeol synthase (GgLUS1)
AB055511.1	*Betula platyphylla*	Lupeol synthase (BPW)
AB181246.1	*Lotus japonicus*	Cycloartenol synthase (OSC5)
AB025968.1	*Glycyrrhiza glabra*	Cycloartenol synthase (GgCAS1)
D89619.1	*Pisum sativum*	Cycloartenol synthase (PSX)
AB009029.1	*Panax ginseng*	Cycloartenol synthase (PNX)
HM623872.1	*Kalanchoe daigremontiana*	Cycloartenol synthase (KdCAS)
AB033335.1	*Luffa cylindrica*	Cycloartenol synthase (LcCAS1)
AB055509.1	*Betula platyphylla*	Cycloartenol synthase (BPX1)
	Maytenus ilicifolia	Friedelin synthase (MiFRS)
	Maytenus ilicifolia	Cycloartenol synthase (MiCAS1)
	Maytenus ilicifolia	Cycloartenol synthase (MiCAS2)

REFERENCES

1 Oliveira, D. M. *et al*. Combined experimental powder X-ray diffraction and DFT data to obtain the lowest energy molecular conformation of friedelin. *Quím. Nova* **35**, 1916-1921, (2012).

2 Guhling, O., Hobl, B., Yeats, T. & Jetter, R. Cloning and characterization of a lupeol synthase involved in the synthesis of epicuticular wax crystals on stem and hypocotyl surfaces of *Ricinus communis*. *Arch Biochem Biophys* **448**, 60-72 (2006).
